# Bleeding-Source Exploration in Subdural Hematoma: Observational Study on the Usefulness of Postmortem Computed Tomography Angiography

**DOI:** 10.3390/diagnostics13132286

**Published:** 2023-07-06

**Authors:** Kazuhisa Funayama, Akihide Koyama, Rieka Katsuragi-Go, Takashi Aoyama, Hiraku Watanabe, Naoya Takahashi, Hisakazu Takatsuka

**Affiliations:** 1 Division of Legal Medicine, Department of Community Preventive Medicine, Graduate School of Medicine and Dental Sciences, Niigata University, Niigata 951-8510, Japan; 2 Center of Cause of Death Investigation, Graduate School of Medical and Dental Sciences, Niigata University, Niigata 951-8510, Japan; 3 Department of Radiological Technology, Graduate School of Health Sciences, Niigata University, Niigata 951-8518, Japan

**Keywords:** postmortem computed tomography, postmortem computed tomography angiography, subdural hematoma, cortical artery, arterial rupture

## Abstract

In a few cases, postmortem computed tomography angiography (PMCTA) is effective in postmortem detection of cortical artery rupture causing subdural hematoma (SDH), which is difficult to detect at autopsy. Here, we explore the usefulness and limitations of PMCTA in detecting the sites of cortical arterial rupture for SDH. In 6 of 10 cases, extravascular leakage of contrast material at nine different places enabled PMCTA to identify cortical arterial rupture. PMCTA did not induce destructive arterial artifacts, which often occur during autopsy. We found that, although not in all cases, PMCTA could show the site of cortical arterial rupture causing subdural hematoma in some cases. This technique is beneficial for cases of SDH autopsy, as it can be performed nondestructively and before destructive artifacts from the autopsy occur.

## 1. Introduction

In recent decades, many research and practical applications of postmortem computed tomography (PMCT) have been carried out, and PMCT is now considered an indispensable diagnostic tool in the field of forensic medicine [1,2]. However, cardiac arrest in cadavers inhibits contrast-enhanced CT, which is routinely used in clinical practice. This limits the application of PMCT in the diagnosis of spontaneous death, particularly coronary artery ischemic disease [1,3,4,5,6,7]. To overcome this limitation, PMCT angiography (PMCTA) has been introduced [8].

In addition to ischemic heart disease [6,9,10,11,12,13,14,15,16,17], PMCTA has been reported for vascular disease (aortic aneurysm rupture [18,19], aortic dissection [14,20,21], peripheral artery aneurysm rupture [22,23], leg varices rupture [24]), tumors [25,26], blunt trauma [27,28,29,30], penetrating trauma [31,32], gunshot wounds [33], and intracranial bleeding [34,35,36,37,38,39].

Of these intracranial hemorrhages, there were three cases of subdural hematoma (SDH), wherein the bleeding source was detected by PMCTA: one case of ruptured saccular aneurysm [36], and two cases of cortical arterial rupture [38]. SDH has been increasing in recent years, especially in the elderly [40], with fatality rates as high as 30%, and, in some settings, as high as 90% [41]. As situations where SDH occurs often involve accidents or crimes [42], sometimes even medical liability [43], the postmortem diagnosis of this disease is becoming increasingly important in the field of forensic medicine, especially in forensic imaging.

Although it is generally considered that SDH is caused by a cerebral contusion or bridging vein rupture as a result of severe head trauma [44,45,46], there is another type of SDH known as “nontraumatic” or “spontaneous” SDH [47,48,49]. Several sources of bleeding caused by minor trauma have been identified in this type of SDH, including cortical artery rupture. Tokoro et al. [50] proposed the following four diagnostic criteria for SDH of cortical arterial origin: (a) no history of major head trauma, (b) no damage to the underlying cortex, (c) no hemorrhagic lesions, such as aneurysm or arteriovenous malformation, around the affected artery, and (d) identification of the arterial rupture at the surgery. Nearly 200 cases of SDH caused by cortical artery rupture have been reported since Scott [51] initially reported it in 1949 [52]. Most reported cases were diagnosed during surgery [38], where the main focus was on treatment, and no histological examination was performed to elucidate the pathology. Autopsies were performed in 40 cases [38,46,48,51,53,54,55,56], with histopathological examination for ruptured cortical arteries performed in only 8 [38,53]. This suggests that histological examination was performed in only 20% of autopsy cases with SDH with a ruptured cortical artery, although the histological examination is one of the main issues of the autopsy.

The rupture hole in the cortical artery is so minute that it can only be seen under microscopic surgery [57,58,59,60]. Alternatively, a thin thread-like spurting bleeding from a cortical artery during surgery alerts to the presence of a rupture [48,51,54]; however, such a tiny rupture of a cortical artery is difficult to notice at autopsy because, of course, arterial bleeding does not occur at autopsy [38]. Consequently, it is assumed that ruptured cortical arteries at the bleeding source of hemorrhage are missed at SDH autopsies [61], resulting in a low histological examination coverage rate for cortical artery rupture [38]. As a result, the accumulation of histopathological searches for cortical artery rupture is insufficient to elucidate its pathogenesis [38,52].

A scheme for detecting cortical artery rupture during autopsy is required to address this issue. Several cases have been reported in which CTA has revealed extravascular leakage of contrast medium from cortical arteries as a clinical imaging finding [62,63,64]. Based on the same findings, PMCTA before craniotomy has been described in autopsy cases to detect cortical artery ruptures [38]. However, the report is limited to two cases, and there is no general discussion on PMCTA in SDH for searching for the bleeding source. Therefore, the usefulness and limitations of PMCTA in SDH remain unknown.

We aimed to determine whether PMCTA is suitable for detecting the source of bleeding in SDH. In this study, we assessed the value and limitations of PMCTA in comparison with the macroscopic findings of an autopsy regarding the detection of cortical arterial rupture.

## 2. Materials and Methods

This observational study was approved by the Niigata University Research Ethics Committee (approval number: 2018-0224 and 2022-0241). The autopsies described in this report were performed in accordance with the legal requirements set forth by the Japanese law, and all procedures followed the principles of the Declaration of Helsinki. The Ethics Committee decided not to seek written informed consent for this study because old forensic autopsy records were needed; however, it was impossible to obtain permission from the subjects’ legal guardians to use that information. This choice was made in accordance with Chapter 5, Section 12-1 (2) (a) and (c) of the “Ethical Guidelines for Medical Research Involving Human Subjects” enacted by the Japanese Ministry of Education, Culture, Sports, Science, and Technology and of the Ministry of Health, Labour, and Welfare [65].

### 2.1. Case Selection

Among the forensic autopsy cases completed in our facility between 2018 and 2022, there were 10 pure SDH cases (79.6 years, range: 62–96 years; 6 men and 4 women). All 10 patients were included in this study, without any exclusion criteria. Based on the duration from head trauma to symptom onset, SDH could be classified as acute (≤3 days), subacute (4–20 days), or chronic (≥21 days) [66]. According to this classification, there were nine acute cases (Case 2–9) and one subacute case (Case 1).

A brief summary of each case is given below.

Case 1: A 95-year-old woman fell in her home, bruised the right side of her face, and sustained a subcutaneous hemorrhage. Thereafter, she presented no further symptoms; however, she died suddenly in her sleep after six days. She was not taken to the hospital, and an autopsy was performed 4 days later. The autopsy revealed right SDH and healing in the subcutaneous hemorrhage around the right cheek, but no other findings of head trauma, including skull fractures, were noted. This case is the same as “Case 2” in the previous literature reported by Funayama et al. [38].

Case 2: An 84-year-old woman fell in a nursing home, bruised the right frontal side of her head, and sustained a subcutaneous hemorrhage; however, she presented no further symptoms. About a month later, she lightly bruised the left side of her head against a wall. She did not show any related symptoms thereafter, but she slipped into a coma 12 h later and was taken to the hospital. She died without craniotomy after 21 h of hospitalization, and an autopsy was performed 6 days later. The autopsy revealed right SDH and no findings of head trauma, including skull fractures or subcutaneous hemorrhage, were noted. This case is the same as “Case 1” in the previous literature reported by Funayama et al. [38].

Case 3: A 77-year-old man fell into a coma after falling down the stairs in his home. He was taken to the hospital and died without craniotomy 8 h after hospitalization. An autopsy was performed 3 days later. The autopsy revealed right SDH and left-sided skull fractures with subcutaneous hemorrhage and laceration.

Case 4: A 96-year-old man was found lying in a toilet of the nursing home. He was laid in his own bed but went into cardiac arrest six hours later. He died during transfer to the hospital, and an autopsy was performed 7 days later. The autopsy revealed right SDH and minor subcutaneous hemorrhages in the right frontal head and the right lip, but no other findings of head trauma, including skull fractures, were noted.

Case 5: A 62-year-old man was found dead in front of his home with blood on his face. He was not taken to the hospital, and an autopsy was performed 5 days later. The autopsy revealed right SDH, a laceration in the right frontal head, and several subcutaneous hemorrhages in the head and face, but no other findings of head trauma, including skull fractures, were noted.

Case 6: A 62-year-old man was found dead in his home. He was not taken to the hospital, and an autopsy was performed 7 days later. The autopsy revealed bilateral SDH and subcutaneous hemorrhage around the left eye; however, no other findings of head trauma, including skull fractures, were noted.

Case 7: A 70-year-old man was found lying in front of his home after 9 h complaining of headache. He returned home but was found dead the next day. He was not taken to the hospital, and an autopsy was performed 6 days later. The autopsy revealed left SDH and several subcutaneous hemorrhages in the head and face, but no other findings of head trauma, including skull fractures, were noted.

Case 8: An 82-year-old woman was found dead in the gutter beside her home. She was not taken to the hospital, and an autopsy was performed 5 days later. The autopsy revealed left SDH and minor subcutaneous hemorrhages in the left and back sides of the head, but no other findings of head trauma, including skull fractures, were noted.

Case 9: An 85-year-old woman, in good health until 2 h before, was found in a coma at her home. She was taken to the hospital. She died without craniotomy 3 days after hospitalization, and an autopsy was performed 3 days later. The autopsy revealed left SDH and subcutaneous hemorrhage in the right and back sides of the head, but no other findings of head trauma, including skull fractures, were noted.

Case 10: An 83-year-old man, in good health until 5 days before, was found dead in his home. He was not taken to the hospital, and an autopsy was performed 7 days later. The autopsy revealed left SDH and a few subcutaneous hemorrhages in the frontal head, but no other findings of head trauma, including skull fractures, were noted.

### 2.2. PMCT and PMCTA

In our facility, we always used a 16-row detector CT scanner (SOMATOM Scope Power Ai Edition; Siemens Medical Solutions, Forchheim, Germany) for non-contrast-enhanced PMCT prior to the autopsy. The following scan and reconstruction settings were used for the head region: field of view, 300 mm; tube voltage, 130 kV; current, 260 mA; feed/rotation, 6.5 mm; pitch factor, 0.7; collimation, 0.6 × 16 mm; and reconstruction interval, 0.6 mm. For the cases with SDH on PMCT, we performed PMCTA during autopsy, following the previously reported methods [38]. Non-ionic water-soluble contrast agent iohexol (OMNIPAQUE^®^ 300 INJECTION; GE Healthcare Inc., Chicago, IL, USA) was diluted with phosphate-buffered saline (PBS) and polyethylene glycol at a ratio of 1:5:10 to produce the contrast medium. We clamped the bilateral external carotid arteries before opening the skull, vertebral arteries in the neck, and contralateral internal carotid and then used a 7-Fr catheter to manually inject 5–25 mL of the contrast medium into the affected side’s internal carotid artery, followed by a head CT. We repeated the injections and imaging until CT images confirmed either extravascular leakage of contrast medium from the cortical arteries or contrast enhancement of the brain parenchyma.

### 2.3. Autopsy to Confirm Cortical Arterial Rupture

After PMCTA, we performed craniotomy, in which the cranial crown was cut using a cutting saw to remove it from the skull, followed by the removal of the SDH as much as possible to expose the brain’s surface. We then injected the PBS into the internal carotid artery of the affected side using the same technique as MPCTA to detect leakage from the cortical artery with PBS perfusion into the cortical artery of the affected side (we did not examine the presence of PBS leakage from the cortical artery of the non-hematoma side via PBS perfusion). If there were macroscopic or histologic findings at or around the site of the leakage during PBS perfusion, providing sufficient evidence of antemortem arterial rupture, such as surrounding hemorrhage, or a focal outer membrane on the inner surface of the dura mater, we decided that the cadaver had cortical arterial rupture; otherwise, we considered it an artifact.

### 2.4. Verification of Artificial Rupture Resulting from PMCTA and Autopsy Technique

We verified the possibility of artificial cortical arterial rupture via intra-arterial injection of the contrast agent used in this study and craniotomy during autopsy as follows: confirmation of the presence of leakage from the cortical arteries via (a) PMCTA using the same procedure in 10 patients with intracranial lesions other than SDH (4 subarachnoid hematomas, 3 intracerebral hemorrhages, 2 cerebral contusions, and 1 epidural hematoma) and (b) PBS perfusion of the cortical arteries following craniotomy using the same procedure in 10 cases without intracranial lesions.

**Figure 1:**
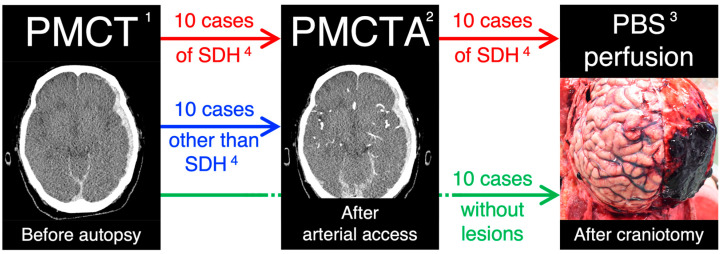
The outline of the methods: (1) postmortem computed tomography, (2) PMCT angiography, (3) phosphate-buffered saline, and (4) subdural hematoma.

Figure 1 presents the outline of the methods.

## 3. Results

### 3.1. Cortical Arterial Rupture and Extravascular Leakage on PMCTA

Table 1 presents a summary of these findings.

In all 10 SDH cases, a total of 15 leakage sites from the cortical artery were confirmed by PBS perfusion after craniotomy during autopsy. A total of 14 sites (1 site in 8 cases, 2 sites in 1 case, and 4 sites in 1 case) were confirmed to be antemortem arterial ruptures based on the autopsy findings. On the affected side of case 1, one leakage site (temporal lobe) was present apart from the prenatal rupture (parietal lobe), which was identified as an artifact due to the complete absence of surrounding hemorrhages.

**Figure 2:**
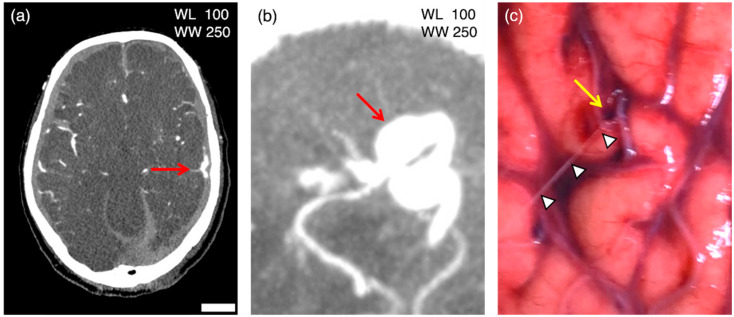
Postmortem computed tomography angiography (PMCTA) images (**a**, axial image; **b**, multi-planar reconstruction image) and macroscopic image of the brain in case 6. PBS perfusion in the left internal carotid artery after craniotomy led to PBS spurting (arrowheads) from the ruptured cortical artery with marginal bleeding (yellow arrow) on the left parietal lobe. PMCTA images show extravascular leakage of the contrast agent (red arrows) at a site consistent with cortical arterial rupture. On the lower right side of (**a**), the bar is equal to 25 mm for (**a**) and 5 mm for (**b**,**c**).

**Figure 3:**
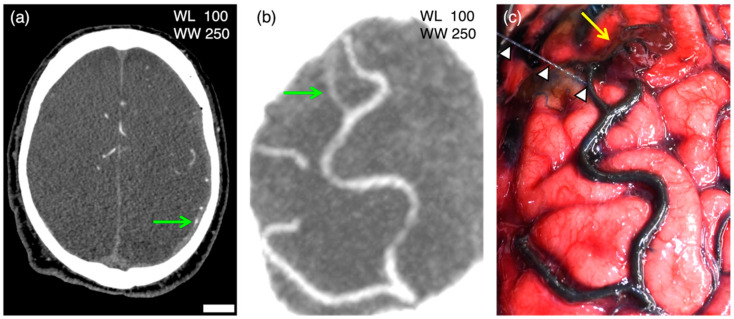
PMCTA images (**a**; axial image; **b**; multi-planar reconstruction image) and macroscopic image of the brain in case 7. PBS perfusion in the left internal carotid artery after craniotomy led to PBS spurting (arrowheads) from the ruptured cortical artery with a neighboring hematoma (yellow arrow) on the left occipital l lobe. PMCTA images show the contrast agent (green arrow) only in the artery at the site consistent with the cortical arterial rupture. On the lower right side of (**a**), the bar is equal to 20 mm for (**a**) and 5 mm for (**b**,**c**).

Of the 14 antemortem ruptures, contrast agent leakage on PMCTA was detected in 9 sites (64.3%) of 6 cases (60%). In the case of four PBS leakages on the right side, contrast agent leakage was identified at every four sites. In the case of bilateral PBS leakages, contrast agent leakage was detected only on the left side (Figure 2), and four of the eight cases with a single PBS leakage had contrast agent leakages at the corresponding sites, whereas the other four cases had no contrast agent leakage, despite the contrast agent being filled in the artery at the site of any PBS leak sites (Figure 3). See Figure S1 for macroscopic images and Figure S2 for CT images of each case.

### 3.2. Artificial Rupture Attributed to PMCTA and Autopsy Technique

**Figure 4:**
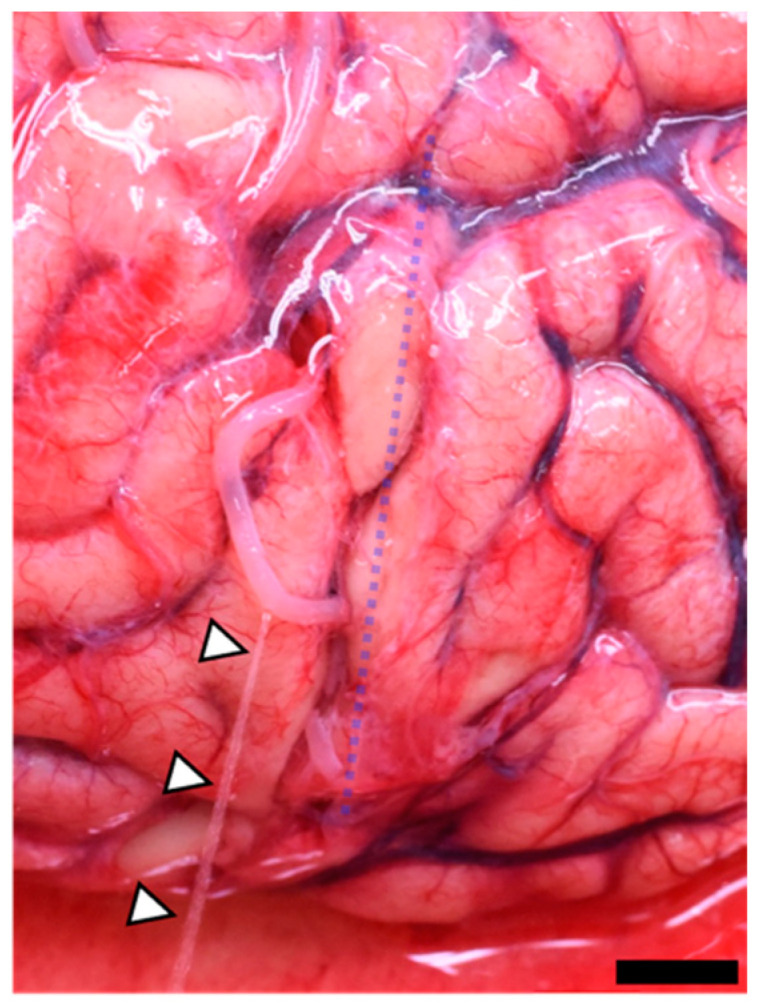
Macroscopic image of the brain without intracranial lesions. PBS perfusion in the internal carotid artery led to PBS spurting (arrowheads) from the cortical artery near the craniotomy line, which is recognizable as an arachnoid tear without marginal bleeding (dotted line).

All 10 cases without SDH had no contrast agent leakage from the cortical arteries on PMCTA. On the other hand, during PBS perfusion following craniotomy during autopsy, 8 in 10 cases without intracranial lesions demonstrated PBS leakages from the cortical artery at a site consistent with the skull cutting line, and all of these leakage sites had no evidence of antemortem arterial ruptures, such as surrounding hemorrhages (Figure 4).

## 4. Discussion

Cortical arterial rupture causes “idiopathic” or “nontraumatic” SDH; however, most of them may be missed at autopsy due to the difficulty in detection [61]. This impedes detailed histopathological investigation, leading to a constantly unknown pathogenesis [38,52]. PMCTA was considered a possible solution to this problem, but, as mentioned at the beginning of this article, this was based on the findings of only two cases. Therefore, in this study, we determined whether PMCTA is useful in detecting cortical arterial rupture in SDH.

PMCTA was able to indicate the location of a cortical arterial rupture before craniotomy during autopsy by detecting extravascular leakage of the contrast agent in more than half of the SDH cases. Contrarily, while PMCTA could not detect all cortical arterial ruptures, post-craniotomy PBS perfusion could accurately identify cortical arterial ruptures. This suggests that PBS perfusion alone is sufficient, and PMCTA is not always necessary for identifying the site of cortical arterial rupture at autopsy in SDH cases. However, a notable advantage of PMCTA is that it can show the site of cortical arterial rupture without making destructive artifacts, unlike the use of a cutting saw, which makes destructive artifacts during autopsy. Previous studies describing the advantages of PMCTA over traditional autopsy have primarily focused on cardiovascular lesions [67] and trauma [68]. This report is novel because it describes the superiority of PMCTA in SDH, an intracranial hematoma.

Only 1 of the 10 SDH cases in this study had destructive artifacts from the cutting saw on the affected side, whereas many cases without intracranial lesions had them. The reason for the difference between the two, in addition to the careful craniotomy in SDH cases, is that the dura mater and arachnoid membrane are potentially attached to each other in normal cases [69], whereas the hematoma separates them in SDH cases, creating a certain distance between the skull bone and brain so that the cutting saw does not easily reach the cortical artery during craniotomy. Even in SDH cases, if the hematoma is small and thin or if the craniotomy technician is inexperienced, the cutting saw may injure the cortical artery, as observed in case 1 of this study.

Furthermore, most cortical arterial ruptures in SDH occur around the Sylvian fissure [70,71], directly beneath the craniotomy cut line. Fortunately, in case 1, the postmortem artifact occurred away from the antemortem arterial rupture, but it is quite possible that they could occur in the same location. This indicates that the slightest finding of a cortical arterial rupture that occurred antemortem could be damaged by postmortem, which may render the subsequent histopathological evaluation difficult. Identification of the site of a cortical arterial rupture before craniotomy allows the cranial crown to be cut, avoiding that site. If the cutting saw creates artifacts, the destruction of antemortem rupture site can be avoided.

Previous studies on PMCTA artifacts have reported that artificial ruptures of an artery [72] or vein [73,74] can occur, although less frequently, due to injection of a contrast agent. PMCTA can be broadly classified into whole-body PMCTA, which contrasts all blood vessels in the body, and focused PMCTA, which contrasts only the arteries perfusing the target organ [7]. The aforementioned artificial vessel rupture occurred in a systemic PMCTA with a pump injection of a contrast agent. On the other hand, we performed a focused PMCTA with manual injection and found no artificial vessel rupture caused by PMCTA. The PMCTA procedure we performed in this study was noninvasive to the vessels of the brain. Conversely, the most frequent artifact of whole-body PMCTA is incomplete contrast filling of cerebral vessels [72,73]. The focused PMCTA technique in this study could fill the contrast agents within the cortical arteries at all rupture sites, preventing the artifact of incomplete contrast filling. However, in nearly half of the cases in this study, there was no extravascular leakage of the contrast agent from the ruptured artery, even though the contrast agent perfused the ruptured site. This is probably because the high cerebral pressure of the SDH blocked the rupture site and did not allow sufficient perfusion pressure for the contrast agent to pass through it. In clinical CT angiography for subdural hematomas, extravascular leakage indicates poor prognosis caused by hematoma enlargement [75,76]. However, the speed of bleeding that increases the hematoma is so minimal that the CT images reportedly suggested slight extravascular leakage 5 min later, rather than immediately after contrast agent injection [77]. This means that extravascular leakage may not be detected on CT unless the flow of the contrast medium is maintained under arterial pressure for 5 min even in the case of an increasing subdural hematoma. The fact that PMCTA may fail to detect extravascular leakage from cortical arterial ruptures in SDH is a limitation of our PMCTA method, which can only allow very brief perfusion of contrast media below arterial pressure (which cannot be accurately measured) to the fully expanded intracranial hypertensive subdural hematoma, as in the fatal case.

Although the mechanism of arterial rupture with minimal traumatic force remains unclear, the hypotheses about the mechanisms proposed so far can be broadly divided into two categories: the first is the inherent vulnerability of the cortical arteries themselves and their interactions with the dura mater [78]. According to the former, the rupture occurs at the bifurcation of a small cortical artery branch, possibly more vulnerable [53,54,79]. Small branches of cortical arteries are sometimes entirely disconnected from their bases, resulting in the “fire hose” rupture [53]. This small branch of the cortical artery would not be visible on PMCTA due to the lack of distal contrast medium perfusion caused by the rupture. Even with medium contrast perfusion, this small branch of the cortical artery is too thin to image with our PMCTA. The latter interaction with the dura indicates the presence of an abnormal structure connecting the cortical artery to the dura, such as a bridging artery [53,54] or adhesions [48,61,80,81]. Even minor forces that shake the brain can tear them off, damaging cortical arteries to cause bleeding [38], but our PMCTA cannot detect such microstructures. Extravascular leakage of the contrast medium from the dura mater can be confirmed in the case of bridging arteries by perfusing the contrast medium from the external carotid artery, which is the dominant artery of the dura mater. Finally, our PMCTA protocol has the limitation that it cannot provide detailed findings on the pathogenesis of cortical artery rupture.

However, as the pathological analysis can be delegated to other methods, such as histological examination and tissue transparency methods, PMCTA is not always required to provide detailed findings on the pathogenesis of cortical artery rupture [38,52]. As previously stated, our PMCTA protocol does not produce destructive artifacts and thus does not interfere with other analysis methods. Given that the missed rupture of cortical arteries precludes histological examination at autopsy, PMCTA would instead facilitate a histological examination by contributing to its prevention. In other words, our PMCTA protocol is highly compatible with autopsy, and, when combined with conventional investigation methods, it is beneficial in elucidating the pathogenesis for SDHs of a cortical arterial origin.

## 5. Conclusions

The site of the cortical arterial rupture that caused SDH was detectable in more than half of the cases (but not all) on PMCTA. This technique is beneficial in cases of SDH autopsy, as it can be performed nondestructively and before destructive artifacts are formed due to the autopsy. Our PMCTA protocol is highly compatible with autopsy and other search methods, and incorporating PMCTA into conventional analysis methods can help us understand the pathogenesis for SDHs of a cortical arterial origin.

## Figures and Tables

**Table 1:** Summary of the results.

No.	Age (Years), Sex	Form of SDH ^1^	Arterial Rupture Site	Contrast Agent Leakage	Total Volume of the Contrast Agent
1	95 Female	Subacute	Right parietal lobe	+	20 mL
2	84 Female	Acute	Right temporal lobe	+	20 mL
3	77 Male	Acute	Right temporal lobe	+	20 mL
4	96 Male	Acute	Right parietal lobe	+	10 mL
5	62 Male	Acute	Right frontal pole Right frontal lobe Right temporal lobe Right parietal lobe	+ + + +	20 mL
6	62 Male	Acute	Left parietal lobe Right parietal lobe	+ -	40 mL (Left) 40 mL (Right)
7	70 Male	Acute	Left occipital lobe	-	65 mL
8	82 Female	Acute	Left parietal lobe	-	65 mL
9	85 Female	Acute	Left temporal lobe	-	55 mL
10	83 Male	Acute	Left parietal lobe	-	65 mL

^1^ Subdural hematoma.

## Data Availability

The DICOM dataset of PMCTA generated and analyzed during the current study is available in Zenodo at https://doi.org/10.5281/zenodo.7870355 (accessed on 27 April 2023). Other data generated or analyzed during this study were included in this published article.

## Supplementary Materials

The following supporting information can be downloaded at: https://www.mdpi.com/article/10.3390/diagnostics13132286/s1, Figure S1: Macroscopic images of cerebral arterial rupture in each case. Figure S2: Postmortem computed tomography angiography images of each case.
