# Supplementary material for: Bleeding-Source Exploration in Subdural Hematoma: Observational Study on the Usefulness of Postmortem Computed Tomography Angiography
Source: Diagnostics (Basel). 2023 Jul 6;13(13):2286. doi: 10.3390/diagnostics13132286 (PMC10340203; doi:10.3390/diagnostics13132286)
Supplement: Supplementary file 1 [file diagnostics-13-02286-s001.zip › diagnostics-2438343-supplementary.pdf]

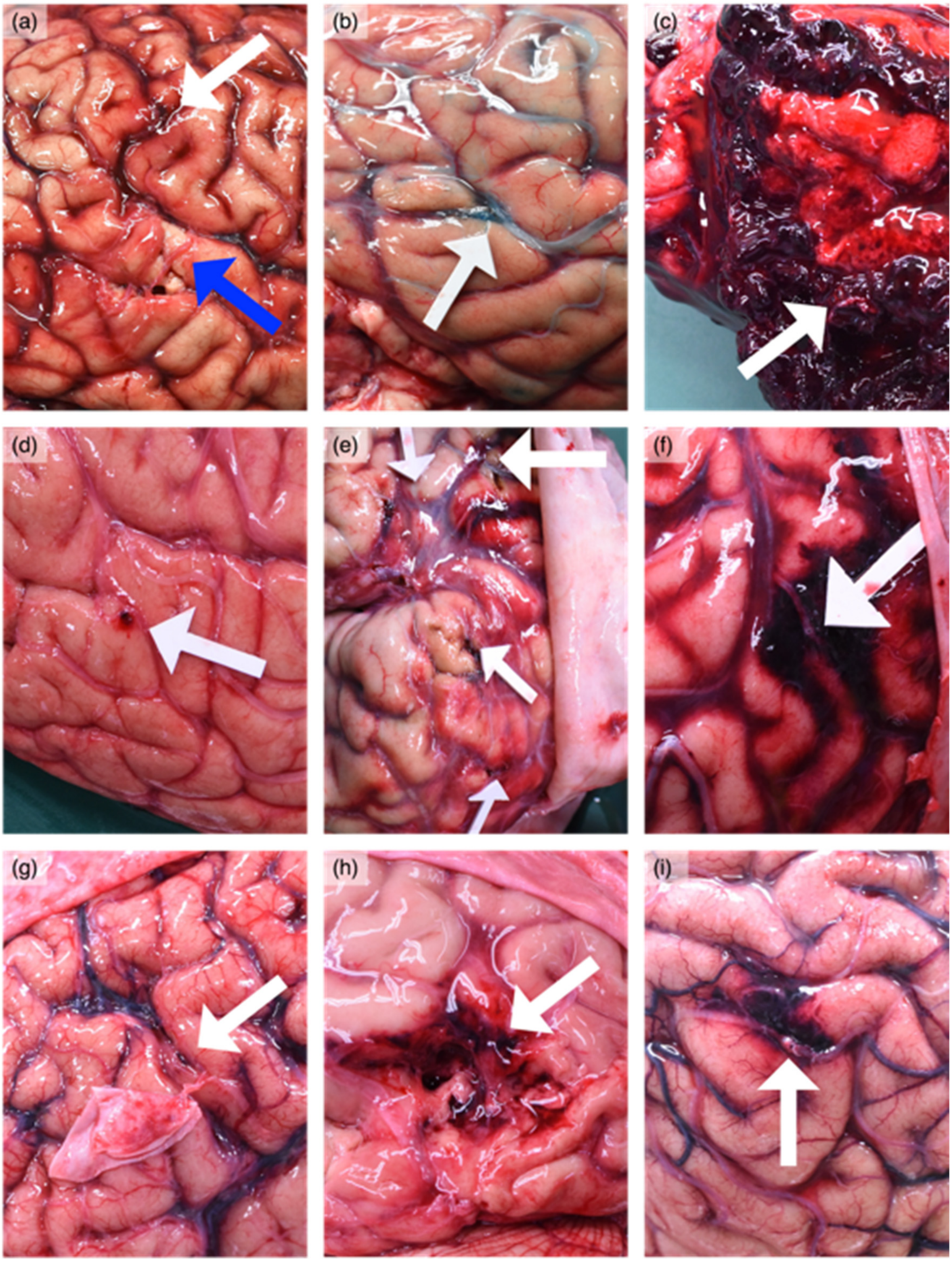

Figure S1. Macroscopic images showing cortical artery rupture in each case. (a) Case 1, (b) case 2, (c) case 3, (d) case 4, (e) case 5, (f) case 6 (right side), (g) case 8, (h) case 9, and (i) case 10. The white arrows indicate arterial rupture; the blue arrow indicates artifact damage.

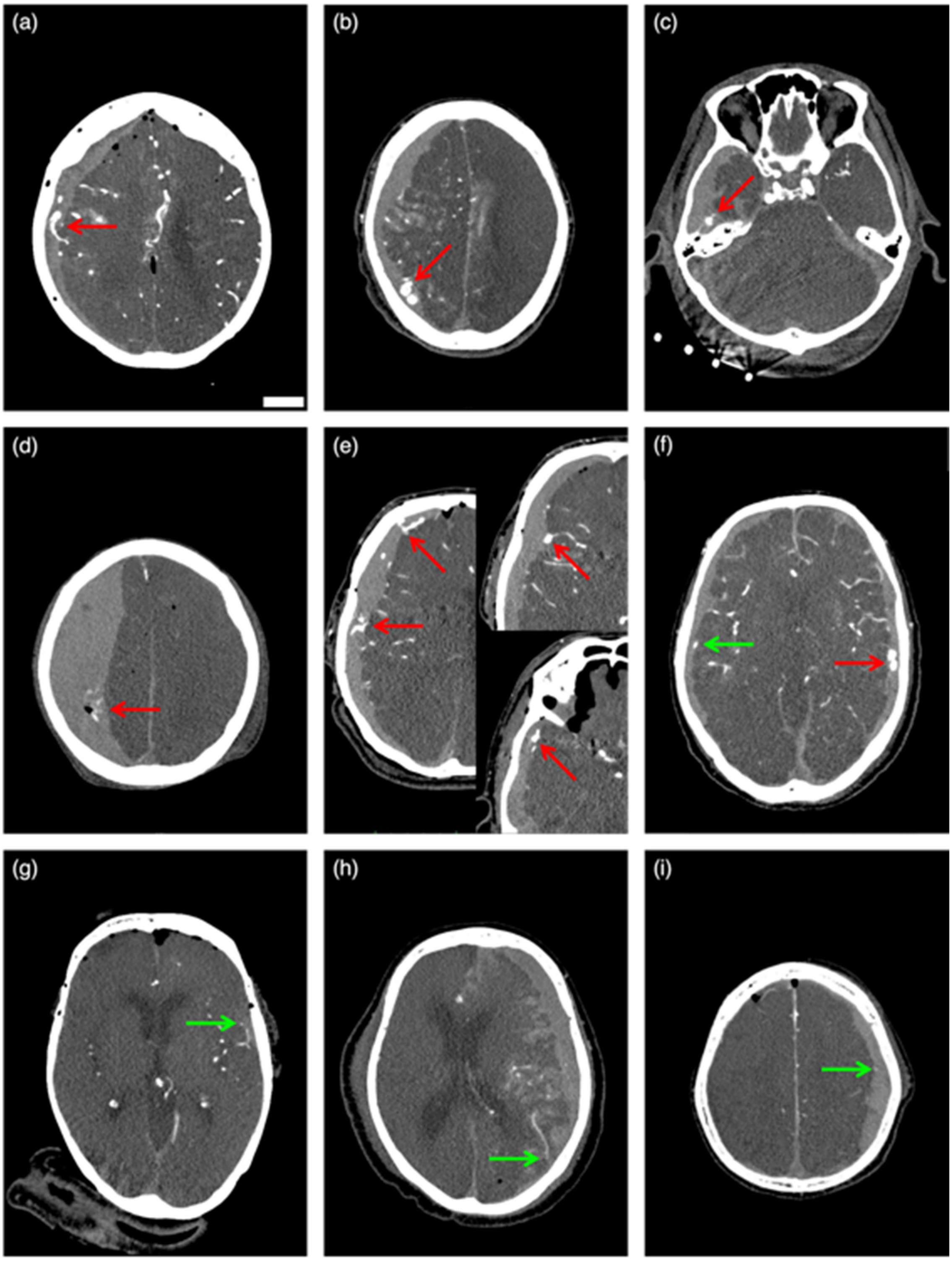

Figure S2. Postmortem computed tomography angiography images of each case. (a) Case 1, (b) case 2, (c) case 3, (d) case 4, (e) case 5 (3 images), (f) case 6, (g) case 8, (h) case 9, and (i) case 10. The white arrows indicate extravascular leakage of the contrast agent; the green arrows indicate no extravascular leakage at the arterial rupture site. Window level is 100, width is 250 and the bar (on the lower right side of (a)) is equal to 25 mm for all. The panels (a,b) are modified from the previous literature reported by Funayama et al. [38].
